# Towards an understanding of C9orf82 protein/CAAP1 function

**DOI:** 10.1371/journal.pone.0210526

**Published:** 2019-01-10

**Authors:** Muhammad Assad Aslam, Mir Farshid Alemdehy, Colin E. J. Pritchard, Ji-Ying Song, Fitriari Izzatunnisa Muhaimin, Ruud H. Wijdeven, Ivo J. Huijbers, Jacques Neefjes, Heinz Jacobs

**Affiliations:** 1 Division of Tumor Biology & Immunology, The Netherlands Cancer Institute, Amsterdam, The Netherlands; 2 Mouse Clinic for Cancer and Aging research (MCCA) Transgenic Facility, The Netherlands Cancer Institute, Amsterdam, The Netherlands; 3 Division of Experimental Animal Pathology, The Netherlands Cancer Institute, Amsterdam, The Netherlands; 4 Department of Chemical Immunology, Leiden University Medical Center LUMC, RC Leiden, The Netherlands; Chang Gung University, TAIWAN

## Abstract

C9orf82 protein, or conserved anti-apoptotic protein 1 or caspase activity and apoptosis inhibitor 1 (CAAP1) has been implicated as a negative regulator of the intrinsic apoptosis pathway by modulating caspase expression and activity. In contrast, an independent genome wide screen for factors capable of driving drug resistance to the topoisomerase II (Topo II) poisons doxorubicin and etoposide, implicated a role for the nuclear protein C9orf82 in delaying DSBs repair downstream of Topo II, hereby sensitizing cells to DSB induced apoptosis. To determine its function in a genetically defined setting *in vivo* and *ex vivo*, we here employed CRISPR/Cas9 technology in zygotes to generate a *C9orf82* knockout mouse model. *C9orf82*^*ko/ko*^ mice were born at a Mendelian ratio and did not display any overt macroscopic or histological abnormalities. DSBs repair dependent processes like lymphocyte development and class switch recombination (CSR) appeared normal, arguing against a link between the *C9orf82* encoded protein and V(D)J recombination or CSR. Most relevant, primary pre-B cell cultures and *Tp53* transformed mouse embryo fibroblasts (MEFs) derived from *C9orf82*^*ko/ko*^ E14.5 and wild type embryos displayed comparable sensitivity to a number of DNA lesions, including DSBs breaks induced by the topoisomerase II inhibitors, etoposide and doxorubicin. Likewise, the kinetics of γH2AX formation and resolution in response to etoposide of C9orf82 protein proficient, deficient and overexpressing MEFs were indistinguishable. These data argue against a direct role of C9orf82 protein in delaying repair of Topo II generated DSBs and regulating apoptosis. The genetically defined systems generated in this study will be of value to determine the actual function of C9orf82 protein.

## Introduction

DNA double-strand breaks (DSBs), that arise for example upon exposure to ionizing irradiation, are very dangerous lesions. If not repaired correctly DSBs can lead to DNA rearrangements and generate gain or loss of function mutations involving oncogenes and tumor suppressor genes, respectively [1, 2]. These mutations can kick-start cancer development [3]. In addition, a delay in DSBs repair or the accumulation of DSBs can trigger DNA damage responses that ultimately may cumulate in the activation of the intrinsic, i.e. death receptor-independent apoptotic pathway [4, 5]. Regardless of their potential to elicit DNA damage responses and the intrinsic apoptotic program, DSBs are critical, physiological intermediates of well-defined biological processes. During replication, topoisomerase II (Topo II) induces DSBs to change DNA topology by relaxing the up winded DNA [6, 7]. Furthermore, DSBs are actively induced in lymphocyte precursors by the RAG recombinase to shape the enormous repertoire of clonally distributed antigen receptors on B and T lymphocytes. These DSBs are central intermediates in the generation of the antigen receptor repertoire of the adaptive immune system [8, 9]. In addition, class switch recombination, also known as antibody isotype switching that enables mature antigen activated B cells to change the immunoglobulin (Ig) heavy chain constant region, is a deletional recombination process between two DSBs induced by the activation induced cytidine deaminase in transcriptionally activated switch regions [10].

In an independent, unbiased genome-wide gene knockout approach, we previously searched for factors capable of driving drug resistance to the topoisomerase II (Topo II) poisons doxorubicin and etoposide, two established longstanding cornerstones of chemotherapy. Keap1, the SWI/SNF complex, and C9orf82 protein were found to drive drug resistance through diverse molecular mechanisms, all converging at the level of DSBs formation and repair. Loss of Keap1 or the SWI/SNF complex was found to inhibit the generation of DSBs by attenuating the expression and activity of topoisomerase IIα, respectively, whereas deletion of *C9orf82* was found to augment subsequent DSBs repair in HAP1 cells and its overexpression delayed DSB repair in MelJuSo melanoma cells [11].

C9orf82 protein, also known as conserved anti-apoptotic protein 1 (CAAP1), or caspase activity and apoptosis inhibitor 1, was first related to the regulation of apoptosis [12]. Knock down of *C9orf82* expression was found to increase Caspase-10 expression and activation and be required for Bid fragmentation and Caspase-9 activation. This study in human A-549 lung and MCF7/casp3-10b breast carcinoma cell lines, which made use of siRNA, suggested an anti-apoptotic function, where CAAP1 was proposed to modulate a Caspase-10 dependent mitochondrial Caspase-3/9 feedback amplification loop [12].

In summary, while C9orf82 protein was initially identified as a negative regulator of the intrinsic apoptosis pathway [12], a subsequent independent study identified C9orf82 protein as a nuclear protein that appeared to control the rate of DSBs repair after exposure to Topo II poison and sensitizes cells to etoposide induced cell death [11]. Accordingly, a *C9orf82* knock down would accelerate DSBs repair and decrease DSBs induced apoptosis, positioning C9orf82 protein not as a direct negative regulator of apoptosis [12] but rather an indirect pro-apoptotic factor of the intrinsic apoptosis shunt [11]. Clearly, to define the function of C9orf82 protein downstream of Topo II induced DSBs and eventually DSBs in general, a genetically defined knockout mouse model and primary cell lines thereof were required to exclude confounders associated with siRNA mediated silencing and using transformed cell lines [11].

In this study, we applied the CRISPR/Cas9 technology to generate a *C9orf82*^ko/ko^ mouse model, and (re)investigated the function of C9orf82 protein in regulating apoptosis and DSBs repair using a genetically defined setting. Our studies failed to confirm previous conclusions on a role of C9orf82 protein in regulating apoptosis and DNA DSBs repair.

## Materials and methods

### Generation of a *C9orf82* knock out mouse model

A *C9orf82* knock out (ko) mouse model was generated by co-injecting zygotes isolated from C57BL/6N mice with *in vitro* transcribed CRISPR/Cas9 encoding mRNA along with guide RNAs (gRNAs) from pX330 plasmid. To inactivate the *C9orf82* locus in the mouse germline (ENSMUSG00000028578), gRNAs flanking exon 2–4 were designed using the crispr.mit.edu tool (Table 1). To distinguish wildtype, heterozygous and homozygous mutants, we established a three primer PCR strategy (Tables 2 and 3). Following the CRISPR/Cas9 procedure, pups born were selected for the knock out allele, backcrossed once onto C57BL6/J and maintained for the desired experimental genotype. Experiments were approved by an independent animal ethics committee of the Netherlands Cancer Institute (Amsterdam, Netherlands) (DEC number 14053) and executed according to Dutch and European guidelines.

**Table 1:** Sequences of gRNAs used to target the *C9orf82* gene.

gRNA	Sequence (5’-3’)
gRNA1	TTAAATAGTGGTATCGCGGCAGG
gRNA2	ATACTATCGTCCACTGACAATGG

**Table 2:** Sequences of primers used to screen for *C9orf82* deleted allele.

Primers	Sequence (5’-3’)
P1	CCGTAGAGGATCTGTGGGTCGGG
P2	TGGAAGGAGACCCATTCAATGCG
P3	GCCAAGCAACACATATTTGATGC

**Table 3:** Primers combinations to detect WT and KO band simultaneously in one PCR reaction.

P1+P2	459 bps	WT Band
P1+P3	367 bps	KO Band

### Primary cell isolation and cell culture

E14.5 embryos resulting from the intercrosses of heterozygous (*C9orf82*^*wt/ko*^) mice were used to isolate primary mouse embryonic fibroblasts (MEFs) and fetal livers. Pre-B cells were generated from single cell suspensions of fetal livers and subsequently grown on irradiated stromal cell line, ST2 feeder cells in complete IMDM medium (Iscoves, supplemented with 8% fetal calf serum (FCS), 50 μM 2-mercapthoethanol, penicillin/streptomycin) containing IL-7. Primary MEFs (2 per genotype) were isolated using Trypsin and cell strainers according to [13, 14] and cultured under low (3%) oxygen condition, with 5% CO2 at 37°C. To immortalize MEFs, primary MEFs were transduced with a lentivirus encoding a p53-specific shRNA [15]. The immortalized MEFs were grown in complete IMDM medium under normal oxygen levels with 5% CO2 at 37°C.

### *C9orf82* cDNA cloning, exogenous expression and qRT-PCR analysis

RNeasy mini (Qiagen) was used to isolate total RNA from wild type MEF for cloning of *C9orf82* and from wild type and *C9orf82*^*ko/ko*^ MEFs for RT-qPCR. The cDNA libraries were synthesized using Invitrogen Superscript III kit and random hexamer primers. *C9orf82* was amplified with high fidelity PfuUltra Hotstart DNA polymerase (Stratagene) using gene specific primers (Table 4). The PCR product isolated from the gel using PCR isolation KIT (Qiagen) was sequenced. After sequencing check, the product was digested with BamHI and Not1 and subsequently cloned into pMX-IRES-GFP vector.

**Table 4:** Sequences of primers used to amplify *C9orf82* expression for subsequent cloning into pMX-IRES-GFP vector.

Primers	Sequence (5’-3’)	
P4	TATCGC***GGATCC***CGCCACCATGACGGGGAAGAAGTCTTC	BamHI site is highlighted
P5	ATACAA***GCGGCCGC***CTACACTGGCTTTTTTATATCACCAG	Not1 site is highlighted

HEK293 T cells were transfected with pMX-IRES-GFP-*C9orf82* and pcl-Eco, according to [16], to produce retrovirus encoding C9orf82 protein. Wild type MEFs were transduced with 48 hrs post transfected supernatant containing retroviral particles and cultured under standard culture conditions. After few days of culturing, MEFs were examined for GFP expression by fluorescent microscope (Zeiss) and live GFP+ cells were sorted by Moflo Astrios (Beckman Coulter).

qRT-PCR was performed on LightCycler 4800II (Roche) using Fast SYBR Green Master Mix (Thermo Scientific). Specific primers (Table 5) were used to amplify GAPDH and *C9orf82* and expression of *C9orf82* was normalized with that of GAPDH.

**Table 5:** Sequences of primers used for measuring relative *C9orf82* expression by qPCR.

Primers	Sequence (5’-3’)	Gene	Target Region
P6	ACTATGCCAGGAACAATTAGAGC	*C9orf82*	Exon 3
P7	ACACAGGTATCTTGCTGACTGA	*C9orf82*	Exon 5
P8	TAGCCCAAAGGAACCCAAAG	*C9orf82*	Exon 6
P9	TCTTGCCCTCATTTCAAGTTCTA	*C9orf82*	Exon 6
P10	CAATGACCCCTTCATTGACC	*GAPDH*	Exon 3
P11	GATCTCGCTCCTGGAAGATG	*GAPDH*	Exon 3

### Class switch recombination

Single cells suspensions were prepared from the spleen of 8 to 10-week-old *C9orf82*^*ko/ko*^ mice and their wild-type littermates. Following erythrocytes lysis, naïve splenic B cells were enriched by the depletion of CD43 expressing cell using biotinylated anti-CD43 antibody (Clone S7, BD Biosciences), BD IMag Streptavidin Particles Plus and the IMag system (BD Biosciences), as described by the manufacturer. To measure their proliferative capacity, naïve B cells were labelled for 10 min at 37°C with 5 μM Carboxyfluorescein succinimidyl ester (CFSE, Molecular Probes) in IMDM medium containing 2% FCS. After washing, cells were cultured in complete IMDM medium at a density of 10^5^ cells/well in 24 well plates. CSR to IgG3 and IgG1 was induced by exposure to LPS (50 μg/ml Escherichia Coli LPS, 055:B5, Sigma) or LPS+rIL-4 (rIL4 20 ng/ml). Four days later, the cells were harvested and stained with CD19-PercpCy5.5 (BD), IgM-APC, and IgG3-PE (LPS cultures) or IgG1-PE (LPS/rIL4 cultures) to determine CSR frequency along with CFSE dilution as an indicator of cell multiplication. IgM-APC, IgG1-PE and IgG3-PE were purchased from Southern Biotech. Data were acquired by flow cytometry (Fortessa, BD) and analyzed using FlowJo software (Version: 10.0.8r1).

### Pre-B cell survival assay

1*10^5^ fetal liver derived pre-B cells from wild type and *C9orf82*^*ko/ko*^ mice were seeded on irradiated ST2 feeder layers in 0.5 ml of complete IMDM medium containing IL-7. For UV (254 nm, UVC irradiation chamber, Dr Grobel UV-Elecktronik, GmbH, Germany) or γ- irradiation (from ^137^Cs source) treatment, 15 min after seeding, the cells were irradiated with different doses and cultured in 1 ml of complete IMDM medium supplemented with IL-7. For Doxorubicin, Cisplatin, Etoposide and MMS, the pre-B cells seeded on irradiated ST2 feeder layers were continuously exposed to different doses of DNA damage agents in 1.0 ml of complete IMDM medium containing IL-7. For determining the survival, pre-B cells were harvested after three days of culture and live (PI negative) cells were counted using FACSArray (Becton Dickinson). Data were analyzed using FlowJo software (Version: 10.0.8r1).

### Colony survival MEFs

MEFs were seeded in 10 cm dishes at a density of 200 cells/dish in complete medium with varying concentrations of Doxorubicin or Etoposide (Pharmachemie). One day later, the medium was removed and replaced with complete medium containing the indicated concentrations of respective drugs. After eight days, the medium containing drug was removed and the cells were washed with PBS and fixed in 5 mL 3:1 v/v Methanol: Acetic acid for 1 h. Following the fixation, colonies were stained by adding 3 ml of 0.3% Coomassie brilliant blue (Merck) solution prepared in H_2_O. After 1.5 h, the staining solution was removed, the dishes were washed with H_2_O and dried overnight. Colonies were counted and the survival of drug-treated cells were normalized for the plating efficiency of untreated cells. Data points represent the mean survival relative to the untreated control cells.

### Western blotting for γH2AX

MEFs proficient or deficient for C9orf82 protein were seeded and next day treated with the indicated amount of etoposide. After an hour, etoposide was washed out and cells were lysed directly in lysis buffer (2% SDS, 10% glycerol, 5% ß-mercaptoethanol, 60mM Tris-HCl pH 6.8 and 0.01% bromophenol blue). Samples were analyzed by SDS-PAGE and Western blotting. Blocking of the filter and antibody incubations (mouse anti-γH2AX (Millipore), mouse anti-actin (Sigma)) were done in PBS supplemented with 0.1% (v/v) Tween and 5% (w/v) milk powder. Blots were imaged using the Odyssey Imaging System (LI-COR).

### Fluorescence activated cell analyses

All the data were acquired by flow cytometry (Fortessa, BD) unless mentioned otherwise and analyzed using FlowJo software (Version: 10.0.8r1). All the antibodies were purchased from BD Pharmingen unless mentioned otherwise.

### 1. T and B lymphocytes

Lymphoid cells from bone marrow, thymus and spleen were isolated from 8–10 weeks old mice. To identify different B and T cell progenitor subsets and mature lymphocytes the cell suspension from bone marrow, thymus and spleen were stained with different mixture of conjugated antibodies (Table 6).

**Table 6:** Antibodies used to study progenitors and mature B and T cells populations.

	Antibodies mixture
Bone Marrow	CD19-APCH7, CD45R (B220)-PacificBlue, IgM-PECy7 (eBioscience), CD117 (cKit)-APC (eBioscience), CD25-PE, IgD-FITC
Thymus	CD3-FITC (Biolegend), CD4-APC, CD8a-PerCp-Cy5.5, CD25-PE, CD44-APCCy7 (Biolegend), TCRß-Pacific Blue (Biolegend)
Spleen	CD3-FITC, CD4-APC, CD8a-PerCp-Cy5.5, CD19-APCH7, CD45R(B220)-PacificBlue, IgD-PE (eBioscience), IgM-PECy7 (eBioscience)

### 2. Annexin-V (Apoptosis assay) and activated caspase-3 measurement

Primary pre-B cells were exposed to different concentrations of DNA damage agents like mentioned in pre-B cells survival assay. After three days of culture, the cells were harvested and stained with Annexin-V according to the manufacturer (FITC Annexin V Apoptosis Detection Kit I, BD) instructions. DAPI was added at the time of measurement to identify different subsets of apoptotic cells. For caspase-3 activation assay, the cells were stained intracellularly with anti-active caspase-3 antibody according to the manufacturer (PE Active Caspase-3 Apoptosis Kit, BD) instructions.

### 3. Cell cycle

Fetal liver derived pre-B cells (1*10^5^) proficient or deficient for C9orf82 protein were exposed to different concentrations of Doxorubicin for 24 hrs. After the treatment, cells were fixed in 70% ethanol and kept at -4°C. Following fixation, cells were treated with RNAse A (0.1 mg/ml, Sigma Chemical Co) for 20 min and then resuspended in PBS containing 5 **μ**g/ml PI. Cells were measured on a FACSCalibur (Becton Dickinson) and the data were analyzed using Flow Jo software (10.0.8r1).

### Statistical analysis

To assess the statistical significance of our data, t-test was performed using Prism 7 (GraphPad).

## Results

### CRISPR/Cas9 mediated inactivation of *C9orf82* in the mouse germline

**Fig 1:**
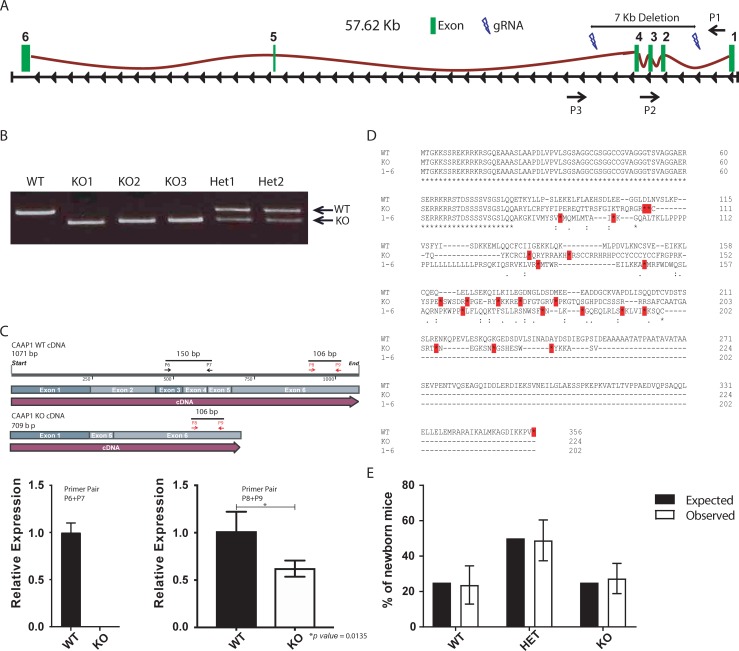
CRISPR/Cas9 mediated inactivation of *C9orf82* in the mouse germline. (A) Schematic presentation of the *C9orf82* locus in the mouse and its CRISPR/Cas9 based inactivation. Cas9 activity targeted by the two gRNAs at the *C9orf82* locus flanking exon 2 and 4 is indicated (blue flashes). The resulting deletion of a 7 kb DNA fragment in the targeted allele can be detected and distinguished from wild type (WT) are indicated by the three primers (black arrows). (B) Genotype PCR to distinguish WT (459 bps) from *C9orf82*^*ko/ko*^ (367 bps) allele. As expected, mice heterozygous for the targeted allele show both bands. (C) Validation of the designed mutation by qRT-PCR. Using qRT-PCR the lack of exons 2,3, and 4 in the mutant cDNA of *C9orf82* was confirming using the primers indicated as black arrows. Stability of truncated *C9orf82* mRNA checked by qRT-PCR using the indicated primers (red arrows) targeting exon 6. (D) *In silico* analysis shows that after exon 2–4 deletion, the remaining transcript either as a result of splicing from exon 1 to exon 5 or exon 1 to 6 contain pre-mature translational stop codons. As a result, after the deletion only the first 82 amino-terminal sequence remains in frame. (E) *C9orf82*^*ko/ko*^ mice were born at Mendelian frequencies. Depicted are the expected and observed frequencies of WT, heterozygous (HET) and homozygous (ko) out of 131 pups born from five breeding pairs (HET x HET).

In mice, the *C9orf82* gene is encoded on the reverse strand of chromosome 4 and is expressed as a single protein-coding mRNA of 2107nt containing an open reading frame 1068nt, i.e. 356aa of an expected molecular weight of 37.83kD. The spliced transcript comprises 6 exons. To functionally characterize C9orf82 protein in mice, *C9orf82* was inactivated by CRISPR/Cas9 genome editing. To avoid potential linkage mutations associated with long-term embryonic stem cell (ES) culturing, we chose for zygote injection. C57BL/6 zygotes were injected with *in vitro* transcribed Cas9 mRNA and two gRNAs flanking exon 2 and 4 of *C9orf82* at chromosomal position 94555956 to 94555978 (gRNA1) and 94548569 to 94548591(gRNA2) (Fig 1A). The next day two-cell stage embryos were implanted into pseudo pregnant foster mothers. In total 33 pups were born and screened by PCR for successful deletion of the targeted 7 kb genomic region using the three primers P1, P2 and P3 (Tables 2 and 3). This strategy allowed direct distinction between wild type, heterozygous, and homozygous *C9orf82* mutant mice (Fig 1B). Four pups were found homozygous for the inactivated *C9orf82* allele (*C9orf82*^*ko*^). The absence of exon 2–4 was validated by Sanger sequencing. Deletion of exon 2–4 was independently confirmed at the cDNA level (Fig 1C). The CRISPR/Cas9 mediated deletion rendered any residual splicing from exon 1 to exon 5 or 6 out of frame. *In silico* analysis suggest that the remaining transcripts will be unstable due to multiple pre-mature translational stop codons rendering the truncated mRNA vulnerable to non-sense mediated decay (Fig 1D). This was confirmed by qRT-PCR using the primers targeting exon 6 that remained present in truncated *C9orf82* mRNA from C9orf82 protein ko mice (Fig 1C). *C9orf82* wildtype, heterozygous and homozygous mice were born at the expected Mendelian frequencies and macroscopic examination of *C9orf82*^*ko/ko*^ mice did not reveal any abnormalities (Fig 1E). These data indicate CAAP1 as a non-essential protein.

### Lack of C9orf82 protein does not impair the development of B and T lymphocytes

**Fig 2:**
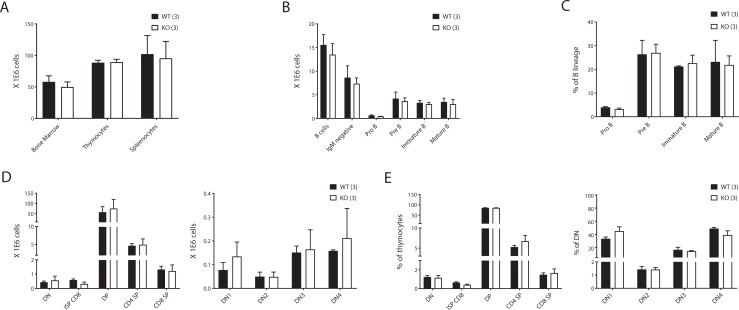
Lymphocytes development in absence of C9orf82 protein. (A) Overall cellularity of nucleated cells from bone marrow, thymus and spleen from *C9orf82*^*ko/ko*^ mice were compared with their wild type littermate controls. (B-C) Developmental subsets of the B cell lineage within the bone marrow. Absolute and relative numbers are shown (see text for specific markers used). (D-E) Developmental subsets of precursor T cells in the thymus. Absolute and relative numbers are compared (see text for specific markers used). Data represent an example of two independent experiments. The results from three wild type mice were compared to three *C9orf82*^*ko/ko*^ mice.

Given the proposed role of C9orf82 protein in controlling/delaying DSBs repair and the generation of developmentally regulated, RAG-induced DSBs early in lymphocyte development led us to study this process in the presence or absence of C9orf82 protein. The cellularity of the bone marrow, thymus, and spleen of *C9orf82*^*ko/ko*^ mice were found indistinguishable from wild-type littermates (Fig 2A).

To explore specific phenotypic alterations in B lymphocyte precursor subsets of the bone marrow, we compared absolute and relative numbers of pro-B cells (CD19^+^, CD45R^low^, CD117^+^, IgM^-^), pre-B cells (CD19^+^, CD25^+^, CD45R^low^, CD117^-^, IgM^-^), immature B cells (CD19^+^, CD25^-^, CD45R^low^, CD117^-^, IgM^+^), and mature B cells (CD19^+^, CD45R^high^, IgM^+^), by staining with a cocktail of IgM, CD19, CD25, CD45R and CD117 specific antibodies each conjugated with a differential fluorophore. Within immature B cells, the developmental subsets were further distinguished as pro B (CD117^+^CD25^-^) and pre-B (CD117^-^CD25^+^). Dead cells were identified as propidium iodide^+^ and excluded from the analysis. B-lineage precursor subsets were found normal in number and frequencies between the genotypes (Fig 2B and 2C).

To extend these analyses to the T cell lineage, thymocytes were stained with a combination of differentially conjugated CD3, CD4, CD8a, CD25, CD44 and TCRß specific monoclonal antibodies. Double negative (DN, CD4^-^/CD8^-^), immature single positive (CD4^-^/CD8^+^/ TCRß^low^), double positive (DP), i.e. CD4^+^/CD8^+^, and mature single positive (SP), i.e. CD4^+^/CD8^-^/TCRß^high^ or CD4^-^/CD8^+^/TCRß^high^ were analyzed. Within the DN compartment the developmental subsets were further distinguished as DN1 (CD44^+^CD25^-^), DN2 (CD44^+^CD25^+^), DN3 (CD44^-^CD25^+^) and DN4 (CD44^-^CD25^-^). Like for B lineage precursors in the bone marrow, the absolute and relative number of T cell progenitor subsets in the thymus did not differ between the genotypes (Fig 2D and 2E).

**Fig 3:**
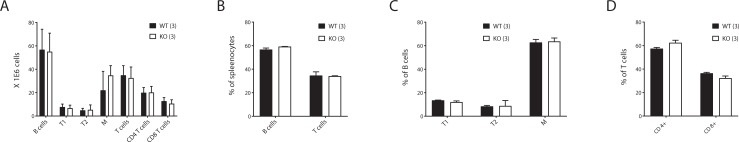
Peripheral (spleen) B and T lymphocytes development without C9orf82 protein. (A) Absolute counts of mature B and T cells with their main subpopulations are indicated (see text for specific markers used). (B-D) Frequencies of mature B and T cells and their specific B cell subsets, and T cell subsets are compared.

To address potential influence of C9orf82 protein deficiency at later stages of lymphocyte development, we investigated the absolute and relative number of mature B and T cell subsets in the spleen. These were distinguished on the basis of CD4^+^ (helper T cells), CD8^+^ (cytotoxic T cells), CD19^+^ (B cells), as well as CD3^-^/CD19^-^ (non-B/T cells). In addition, the proportional size of different subsets of mature B cells as defined by IgM and IgD surface expression (T1; IgM^+^IgD^-^, T2; IgM^+^IgD^+^ and M; IgM^-^IgD^+^) were also included in the analyses. Like in primary lymphatic organs, the cellularity and composition of mature lymphocyte subsets in the spleen were found similar between the *C9orf82*^*ko/ko*^ and wild type mice (Fig 3A–3D). Herewith, we conclude that the inactivation of *C9of82* has no critical impact on B or T cells from early to mature stage of development.

### C9orf82 protein does not influence class switch recombination (CSR) and proliferation of B cells *in vitro*

**Fig 4:**
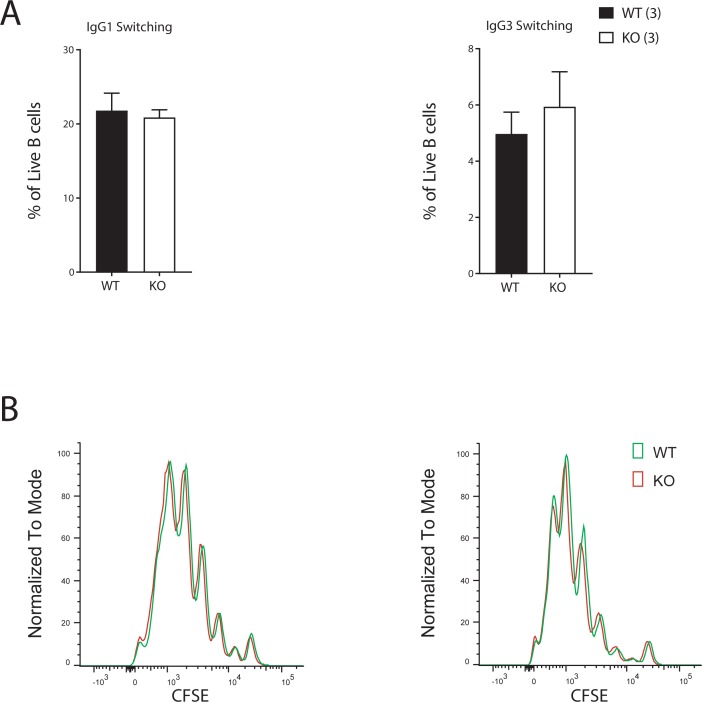
Class switch recombination and proliferative potential of B cells lacking C9orf82 protein. (A) The class switch recombination potential as determined *in vitro* by switching of antigen inexperienced B cells to IgG3 and IgG1 after 4 days of exposure to LPS alone or LPS+IL-4, respectively is compared between wild type and *C9orf82*^*ko/ko*^. (B) Proliferative potential of antigen inexperienced B cells after 3 days of exposure to LPS alone or LPS+IL-4 was measured by CFSE dilution and compared between the genotypes (a representation of 3 mice per genotype is shown).

Previous reports suggested a role of C9orf82 protein in regulating DSBs repair [11]. This observation let us to hypothesize that C9orf82 protein may influence class switch recombination (CSR), a recombination process that strongly depends on the generation of two concomitant DSBs in active switch regions of the Ig heavy chain locus/loci. To investigate whether the absence of C9orf82 protein influences CSR, CSR frequencies of CFSE labelled, naïve splenic B cells (CD43^-^) from *C9orf82*^*ko/ko*^ and wild type littermates were compared. Antigen inexperienced B cells from *C9orf82*^*ko/ko*^ and wild type mice showed similar percentage of switching to IgG3 and IgG1 after being exposed to LPS alone or LPS and IL4, respectively (Fig 4A). Furthermore, their proliferative potential as measured by CFSE dilution did not differ between the genotypes respectively (Fig 4B). Apparently, C9orf82 protein deficiency does not alter the proliferative capacity and CSR potential.

### DNA damage sensitivity is not determined by C9orf82 protein

**Fig 5:**
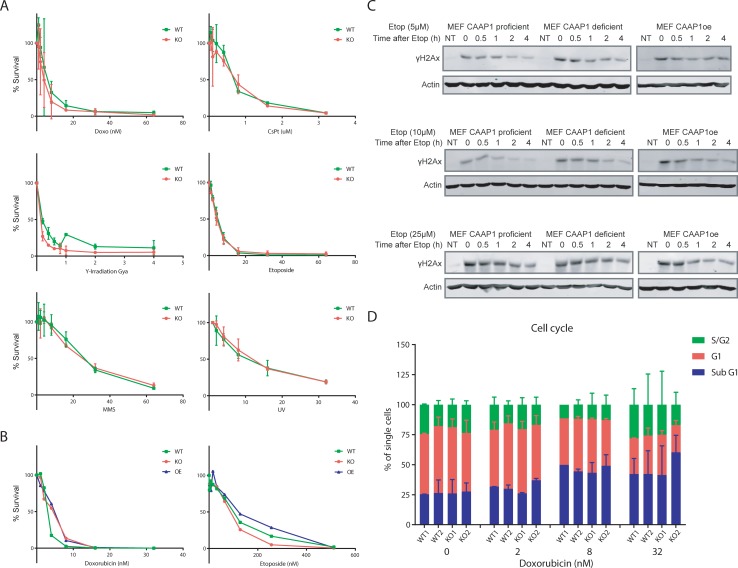
Sensitivity of primary and transformed cells to DNA damaging agents in the presence and absence of C9orf82 protein. (A) Relative survival of pre-B cells from wild type and *C9orf82*^*ko/ko*^ mice upon exposure to increasing doses of UVC, cisplatin, MMS, γ-irradiation, doxorubicin and etoposide was analyzed. (B) Colony survival rate of transformed MEFs from wild type and *C9orf82*^*ko/ko*^ mice upon exposure to Doxorubicin and Etoposide was compared. (C) Recovery from DNA damage response. The recovery rate from γH2AX generated in Tp53kd transformed MEFs in response to etoposide induced DSBs are compared. (D) Cell cycle analysis and relative contribution of sub-G, G1, and S/G2 of pre-B cells from the indicted genotypes.

The role of C9orf82 protein in delaying Topo II poisons induced double stranded DNA repair, was suggested on the basis of the kinetics of γH2AX resolution [11]. Independently, C9orf82 has been implicated as a pro-survival protein in response to intrinsic apoptotic signals. This led us to investigate the role of C9orf82 protein in controlling the sensitivity to DNA damage. Consequently, this would alter cell survival upon exposure to different DNA damaging agents. As a measure of DNA damage sensitivity, we analyzed the survival of primary pre-B cells to increasing doses of DNA damage inducers. These included: Cisplatin, methyl-methane sulfonate (MMS), Doxorubicin, Etoposide, UV-C, and gamma radiation. Strikingly, wild type and C9orf82 protein deficient cells were found to be equally sensitive to damages caused by replication blocking genotoxic agents, *i*.*e* DNA crosslinks induced by UV-C and cisplatin, and DNA alkylation induced by MMS. Furthermore, *C9orf82*^*ko/ko*^ and wild type pre-B cells were not differentially sensitive to double strand breaks induced by γ-irradiation, Doxorubicin and Etoposide (Fig 5A).

Independently, mouse embryo fibroblasts (MEFs) transformed by a TP53kd were also tested for their sensitivity against the Topo II poisons, Doxorubicin and Etoposide. This also included retrovirally transduced MEFs overexpressing C9orf82 protein. The colony survival rate of MEFs from wild type was indistinguishable from that of C9orf82 protein deficient and over expressing MEFs (Fig 5B). Analysis of the results from two independent systems measuring the sensitivity of pre-B cells as well as transformed MEFs upon DNA damage helped us to conclude that C9orf82 protein does not confer resistance to DNA damaging agents.

### Rate of DNA repair is not influenced by C9orf82 protein

As mentioned earlier, C9orf82 protein has been associated with delaying the repair of Topo II poisons induced DSBs, measured by the kinetics of γH2AX resolution, a surrogate marker for DNA damage [11]. If so, C9orf82 protein deficient MEFs are expected to show quicker resolution of γH2AX upon etoposide exposure, a Topo II poison. To test this hypothesis, C9orf82 protein deficient and proficient MEFs were exposed to various concentrations of etoposide. Following 1 hour of incubation, the cells were washed to remove etoposide. After the indicated time points, cells were lysed and analyzed by western blot for the expression of γH2AX. This analysis demonstrated that at various time points and concentrations of etoposide, the resolution of γH2AX did not differ between these settings. Furthermore, the expression of γH2AX was not different between the C9orf82 protein deficient and overexpressing MEFs (Fig 5C). Here again, in contrast to the previous study [11], our results show that in MEFs, C9orf82 protein does not influence the rate of DNA repair.

Apparently, two independent systems measuring the survival of primary cells as well as primary transformed cells upon DNA damage contradict previous findings in cell lines, suggesting that *C9orf82*^*ko/ko*^ cells repair DNA damage faster than wild type and over expressing cells [11], which should have led to better survival. We conclude that in primary cells, expression of C9orf82 protein does not affect cell survival upon DNA damage suggesting that it has no critical role in the DSBs repair process.

### C9orf82 protein deficiency does not alter cell cycle in primary B cells

To allow DNA synthesis to proceed, the positive supercoils must be relaxed. In eukaryotes this is accomplished by type I and type II topoisomerases [6]. This led us to analyze the impact of C9orf82 protein deficiency on the cell cycle in primary cells upon exposure to the Topo II poison doxorubicin. Two independent fetal liver derived primary pre-B cell cultures from C9orf82 protein proficient and deficient were exposed to increasing dose of Doxorubicin. Following ethanol fixation, cells were stained with PI to measure DNA contents by FACS. Cell cycle analyses identified different phases of cell cycle including sub G1, G1 and S/G2 (Fig 5D). Our results showed that cell cycle profile from C9orf82 protein deficient cells was very similar to C9orf82 protein proficient cells. This experiment led us to conclude that C9orf82 protein does not control the cell cycle. ​

### C9orf82 protein does not control intrinsic apoptosis in primary cells

**Fig 6:**
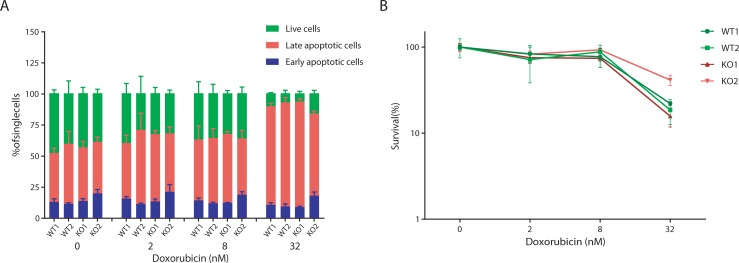
Apoptotic responses to Doxorubicin in the presence and absence of C9orf82 protein. (A) The percentage of live, early apoptotic cells (Annexin V^+^), and late apoptotic cells (Annexin V^+^, DAPI^+^), were very similar between the polyclonal pre-B cell cultures established from the fetal livers of two independent E14.5 embryos per genotype. (B) Independent presentation of live cells as percentage of all nucleated cells.

C9orf82 protein has been reported as a negative regulator of the intrinsic pathway of apoptosis [12]. In this case, DNA damage induced by Doxorubicin, a topoisomerase II inhibitor is expected to increase apoptosis of C9orf82 protein deficient cells. To test this concept, wild type and *C9orf82*^*ko/ko*^ fetal liver pre-B cells were exposed to different concentrations of Doxorubicin for 72 hrs and then co-stained with DAPI and Annexin V for subsequent FACS measurement. Early and late apoptotic cells were distinguished as Annexin V^+^ DAPI^-^ and Annexin V^+^ DAPI^+^, respectively. This analysis revealed that C9orf82 protein deficient pre-B cells were as sensitive as the wild type controls (Fig 6A). Furthermore, the percentage of cells survival also remained indistinguishable in this setting (Fig 6B). In contrast to a previous study [12], our result led us to conclude that C9orf82 protein has no critical role in regulating cell death in primary cells.

### C9orf82 protein deficiency does not elevate active caspase-3 level in primary B cells

**Fig 7:**
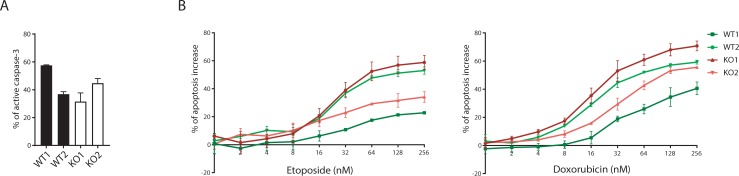
Active caspase-3 level in C9orf82 protein proficient and deficient cells in response to Doxorubicin and Etoposide. (A) The percentage of active Caspase-3 without prior exposure to Doxorubicin and Etoposide was compared between the polyclonal pre-B cell cultures established from the fetal livers of two independent E14.5 embryos per genotype. (B) Relative contribution of Doxorubicin and Etoposide exposure to the level of active Caspase-3 was analyzed between the fetal liver derived pre-B cell cultures from two independent E14.5 embryos per genotype.

C9orf82 protein has been identified as a negative regulator of caspase-mediated apoptosis in lung and breast carcinoma cell lines [12]. Based on this report we hypothesized that the primary cells lacking C9orf82 protein would show higher levels of active caspase-3. To test our hypothesis, we checked the active caspase-3 level by FACS in the C9orf82 protein proficient and deficient fetal liver derived primary B cells. In contrast to the previous report, we found that the level of active caspase-3 was not significantly different between the genotypes. Furthermore, the level of activated caspase-3 was also measured after exposure of C9orf82 protein proficient and deficient primary cells to different concentrations of etoposide and doxorubicin for 16 hrs. This analysis revealed that the increase in the activated caspase-3 level upon increasing concentrations of Etoposide and Doxorubicin was independent of the expression of C9orf82 protein (Fig 7). These results are in line with our findings regarding the absence of correlation of C9orf82 protein expression with DNA damage sensitivity and intrinsic apoptosis, and this led us to conclude that C9orf82 protein does not influence the activity of caspase-3 in primary cells.

## Discussion

A primary study addressing the function of the highly conserved C9orf82 protein in human cell lines implicated an anti-apoptotic function [12]. Independently, we discovered C9orf82 protein in an unbiased genome-wide screening approach, aiming at the identification of potential factors conferring resistance to Topoisomerase II poisons, *i*.*e*. Etoposide and Doxorubicin. Our data, implicated that the resistance to Topo II inhibitors in absence of C9orf82 protein is due to its potential to delay DSBs repair incurred by TOPO-II. Subcellular analysis revealed that C9orf82 protein is a nuclear protein, arguing against a direct control of cytosolic Caspases, as reported [12]. An anti-apoptotic function was further counter argued by another study, where two Sézary syndrome patients with a mono or bi-allelic deletion of *C9orf82* did not display increased apoptosis [17]. Hence, two primary, independent research studies addressing the function of C9orf82 protein report opposing results.

In view of many inconsistencies and the potential confounders in these studies, we opted for the generation of a genetically well-defined *C9orf82*^*ko/ko*^ mouse model. This enabled us to explore the function of C9orf82 protein in mice and primary cell cultures established from mouse embryos. A homozygous deletion of *C9orf82* in mice did not show any macroscopic or histological abnormality and mice were born at expected mendelian frequencies. This indicates that C9orf82 protein is a non-essential protein.

Given the previous insights on the role of C9orf82 protein in delaying DSB repair, and the dependence of early lymphocytes on Rag-induced DSB repair during V(D)J recombination, we first investigated the impact of the *C9orf82* ko on early lymphocyte development. Our results showed that lymphocytes development was not affected by the absence of C9of82 protein. Likewise, *in vitro* analysis of IgH chain class switch recombination (CSR) in mature B cells excluded a critical role of C9orf82 protein in this DSB-repair dependent process.

As we identified C9orf82 protein as a factor conferring resistance to Topo II inhibitors, we first wanted to validate our previous findings in tumor cell lines to two independent, well-established primary and secondary cellular systems, i.e. primary pre-B cell cultures and immortalized MEFs (TP53kd), respectively. Testing the DNA damage sensitivity of C9orf82 protein proficient and deficient cells, including MEFs that overexpress C9orf82 protein against various DNA damaging agents we observed, that loss of C9orf82 protein does not confer resistance to DNA damaging agents, including Topo II inhibitors.

As Topo I and II play a crucial role in DNA unwinding during replication and thereby influences the cell cycle [6], we reasoned if C9orf82 protein confers resistance to Topo II inhibitors, cell cycle may be affected in the absence of C9orf82 protein. Our results from un-exposed and exposed primary pre-B cells cultures, proficient and deficient for C9orf82 protein, indicated that the C9orf82 protein does not regulate the cell cycle. Furthermore, while our previous report in tumor cell lines suggested that C9orf82 protein delays DSB repair as deduced from γH2AX resolution upon etoposide exposure, our present results obtained from wild type, C9orf82 protein deficient and MEFs over expressing C9orf82 protein did not show any genotype/phenotype changes. In line with the conclusions drawn from T and B cells development and CSR data, the results from γH2AX resolution experiment strongly argue against a role of C9orf82 protein in regulating DSBs repair.

To test anti-apoptotic potential of C9orf82 protein as suggested by [12], C9orf82 protein deficient and proficient primary pre-B cells were exposed to doxorubicin. Subsequent FACS analysis using Annexin-V and DAPI staining showed that C9orf82 protein does not control intrinsic apoptosis in primary cells. In line with these results, measuring the fraction (percentage) and amount (median fluorescence intensity; MFI) of activated caspase-3 in primary pre-B cells exposed to doxorubicin and etoposide, indicated that absence of C9orf82 protein did not alter the level of caspase-3 activation in primary cells, further arguing against an anti- apoptotic role.

The evolutionary conserved nature of *C9orf82* and the lack of any known related protein family members argue against transspecies differences and compensatory back-up mechanisms in the absence of C9orf82 protein, respectively. The discrepancies between our primary study in human HAP1 and MelJuSo cell lines and mouse knock out system likely relate to differences in the cell type rather the strategy to knock out *C9orf82*. We like to note, that our initial functional analysis of *C9orf82* inactivation was limited to the characterization of the HAP1 *C9orf82* ko clone whereas our analysis of triple negative breast cancer patients failed to reveal any correlation between C9orf82 protein expression and the response to the treatment with doxorubicin-containing regimen [11]. It remains to be determined if our primary phenotype in HAP1 leukemic cells is cancer specific.

Future attempts that aim to determine the actual function of C9orf82 protein may benefit from an unbiased hypothesis generating approach, like combined co-immunoprecipitation/mass spectroscopy studies. The identification of specific binding partners of C9orf82 protein may pinpoint the key biological pathway(s) controlled by C9orf82 protein. We propose that the term CAAP1 for the protein encoded by *C9orf82* should be omitted as it is misleading and be reconsidered once the exact function has been identified.

In conclusion, although our data failed to confirm previous findings, this study has generated a valuable, genetically well-defined mouse model and cell lines thereof. These will enable the scientific community to explore the actual role of C9orf82 protein at the systemic, organ, cellular, and molecular level *in vivo* as well as *in vitro* settings, where confounding issue scan be excluded.
